# Paraneoplastic brainstem encephalitis in a patient with exceptionally long course of a metastasized neuroendocrine rectum neoplasm

**DOI:** 10.1186/1471-2407-14-691

**Published:** 2014-09-22

**Authors:** Michael Boch, Anja Rinke, Peter Rexin, Maria Seipelt, Dörte Brödje, Marvin Schober, Thomas M Gress, Patrick Michl, Sebastian Krug

**Affiliations:** Department of Gastroenterology, Endocrinology and Metabolism, Philipps-University Marburg, Baldingerstrasse, 35043 Marburg, Germany; Institute of Pathology, Philipps-University Marburg, Marburg, Germany; Department of Neurology, Philipps-University Marburg, Marburg, Germany; Institute of Laboratory Medicine and Pathobiochemistry, Molecular Diagnostics, Philipps-University Marburg, Marburg, Germany

**Keywords:** Neuroendocrine rectum neoplasm, TACE, Paraneoplastic syndrome, Anti-Ri-antibody, ANNA-2

## Abstract

**Background:**

Paraneoplastic neurological syndromes (PNS) have frequently been described in patients with lung or breast cancer. However, some reports also described a correlation to carcinoid tumors, probably triggered via the excessive release of hormones.

**Case presentation:**

We report the case of a 40-year-old woman that was diagnosed with a neuroendocrine neoplasm (NEN) of the rectum and multiple synchronous liver metastases ten years ago. She initially responded well to transarterial chemoembolization (TACE), resulting in prolonged disease stabilization. However, ten years after initial diagnosis the patient developed unspecific neurological symptoms that could not be classified by standard neurological diagnostic work-up. Special laboratory analysis revealed a high titer of anti-Ri (ANNA-2), a well-characterized antibody that is associated with paraneoplastic neurologic syndromes. The patient’s symptoms improved markedly after a 5-day-course of high-dose glucocorticoid therapy. To our knowledge, this is the first report of a Ri-positive PNS in a patient with hormone-negative rectal NEN.

**Conclusion:**

PNS can complicate the patient’s clinical course, response to treatment, impact prognosis and even be interpreted as metastatic spread. However, owing to their rarity, the knowledge of these syndromes is very helpful in order to be able to provide evidence-based diagnostic and therapeutic approaches.

## Background

Neuroendocrine Neoplasms (NENs) of the rectum have been increasing in incidence over the last decades and now comprise 16% of all NENs [1]. Most rectal NENs are localized at diagnosis with acceptable 5-year overall survival rates of approximately 90%. However, regional and distant disease is associated with a dramatically impaired outcome. Factors influencing survival included tumor size, histology, proliferation index, depth of invasion and lymphvascular invasion [2]. Localized tumors that are small (T1 and <1-2 cm of size) can be managed with endoscopic resection. For advanced disease the benefit of radical surgery remains to be elucidated [3]. So far, no published data on treatment outcomes for the metastatic situation are existing, therefore, multidisciplinary treatment options should be included into considerations [2].

Paraneoplastic neurological syndromes (PNS) are mainly associated with lung cancer, gynecological tumors and hematological diseases. The expression of antigens by various neoplasms lead to antibody formation that may induce an intrathecal inflammatory process leading to distinct neurological symptoms [4, 5]. Moreover, specific antigen recognition of antibodies in neuronal tissue induces characteristic neurological symptoms. There are few cases describing concomitant neurological symptoms in patients with neuroendocrine neoplasms, however, mostly due to excessive hormone release and seldomly correlated to positive antibodies [6]. This report represents the first case of an anti-Ri positive paraneoplastic brainstem encephalitis in a patient with exceptionally long course of a metastasized neuroendocrine rectum neoplasm.

## Case presentation

**Figure 1:**
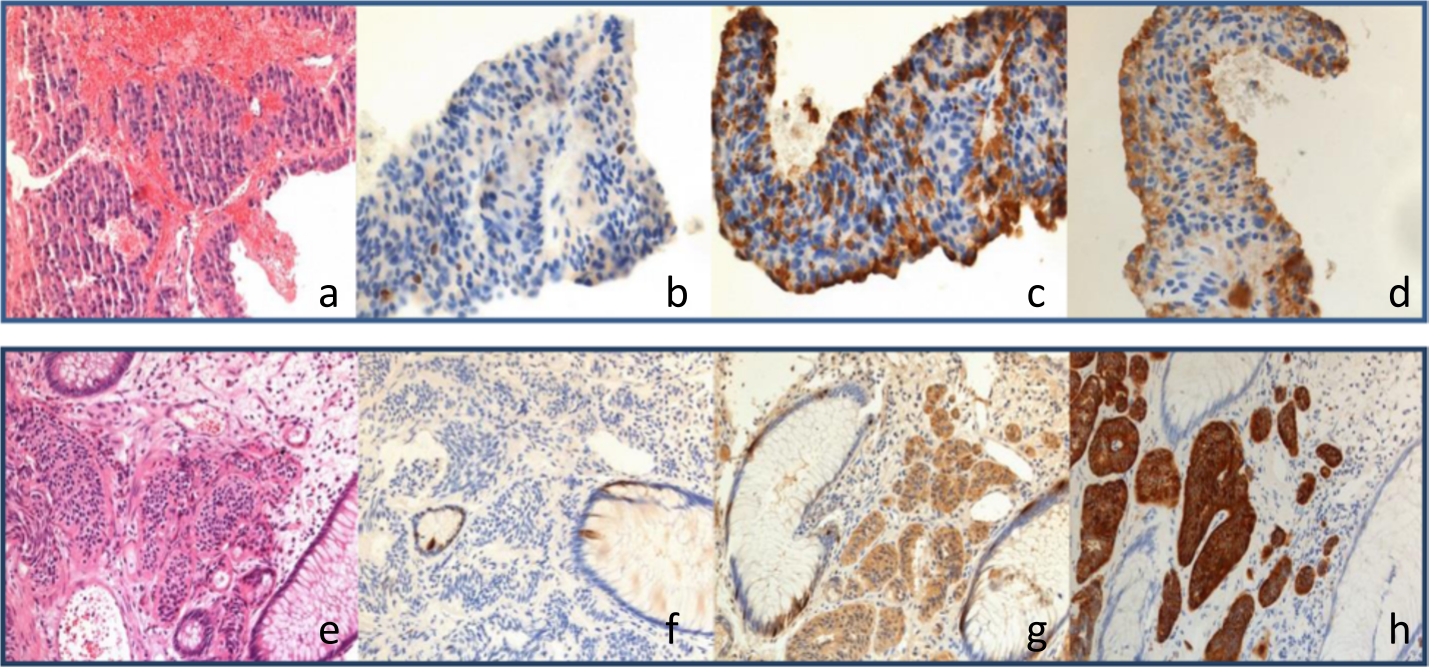
**Immunohistochemical characteristics of primary and liver metastases.** The initial liver biopsy shows the characteristic cell formation pattern of neuroendocrine tumor cells of solid nests of regular cells with broad eosinophilic cytoplasm and small inconspicuous nuclei. Mitotic cells are absent **(a)**. The proliferation rate is very low, Ki67 marks less than 1% of the tumor cell nuclei **(b)**. Furthermore, the strong immunohistochemical reaction for chromogranin A and synaptophysin is a solid proof for neuroendocrine differentiation **(c and d)**. Likewise the rectum polyp exhibits the same histological **(e)** and immunohistochemical features including low proliferation **(f)** and marked reaction for the neuroendocrine markers chromogranin A and synaptophysin **(g and h)**.

A 40-year old woman was referred to our clinic in 2003 after detection of multiple lesions in both hepatic lobes during routine ultrasound examination. The patient did not report weight loss, night sweats or fever. She had no flush symptoms or diarrhea, only a slight postprandial discomfort in the right upper quadrant. Apart from a moderate hepatomegaly, physical examination was unremarkable. Routine laboratory findings were within normal limits without indication of liver disease or advanced malignancy. Likewise, chromogranin A, serotonin and 5-HIAA were within normal range. Sonographically the lesions were of high echogenicity with a hypoechoic halo. The largest lesion found in segment VI had a diameter of 6.8 cm. Multiple additional hepatic lesions were spread throughout both lobes with a size of approximately 1 cm. A CT-guided core biopsy was performed to establish histopathological diagnosis. Histological analysis of the biopsy material showed a G1 neuroendocrine neoplasm (NEN) with a low proliferation rate (Ki-67 < 1%) (Figure 1a-d).

For tumor staging, abdominal imaging by MRI and a chest CT scan were performed which confirmed multiple bilobular hepatic lesions with no option for complete surgical removal. Somatostatin receptor scintigraphy (SRS) showed a positive receptor status of the hepatic lesions. Colonoscopy identified the primary tumor in the rectum: 5 cm from the anus a polyp with 1 cm diameter was found and endoscopically removed by snare biopsy. Microscopical analysis confirmed a G1 NEN with similar histological appearance as seen in the hepatic metastases (Figure 1e-h). Due to the positive somatostatin (SMS) receptor status treatment with octreotide was started. 6 months later, however, a restaging revealed progression of the liver metastases. As there was no evidence of extrahepatic manifestation we decided to perform a transarterial chemoembolization (TACE) of the hepatic lesions. The patient received in total 7 TACE interventions without complications over a period of 2 years. Over a follow-up period of 7 years without further anti-cancer therapy up to the present, MRI revealed stable disease according to RECIST criteria.

**Figure 2:**
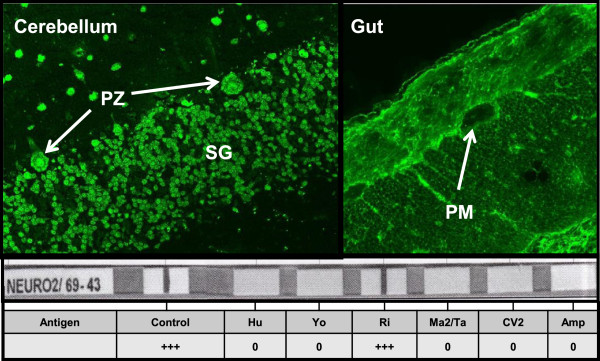
**Validation of Ri-antibodies via IIFT and immunoblot assay.** Characteristic granular fluorescence staining of all neurone nuclei of the grey matter of primate cerebellum (upper left**)**. (SM = stratum moleculare; SG = stratum granulosum; PZ = Purkinje cells). Peripheral neurons of the plexus myentericus (PM) revealed no binding of Ri-antibodies (upper right). High positive reaction with the recombinant Ri-antigen (Nova1) in Lineblot (lower panel) confirmed the IIFT result. No reactivity was observed for anti-Hu, anti-Yo, anti-Ma2/Ta, anti-CV2 and anti-amphiphysin.

In 2013, however, the patient presented to the general practitioner with hoarseness as well as tenderness on palpation and stabbing pain in the right lower jaw, the mandibular joint and the right part of the tounge. During the course of several weeks, additional symptoms like palsy of the tongue and dysarthria emerged and the patient was put on an empiric antibiotic treatment with a cephalosporine. With increasing numbness of the tongue and on the inner surface of the cheek, pain of the tongue, worsening dysarthria and dysphagia the patient presented to the emergency department of our hospital. A thorough neurological examination including cranial computed tomography, MRI with MR-angiography and a time of flight (TOF)-imaging as well as ENT examination was not instrumental to establish a diagnosis. However, a lumbar cerebrospinal fluid (CSF) puncture revealed a pleocytosis (19 cells/μl) as well as an intrathecal protein synthesis (IgG), both in accordance with an inflammatory process in the brain. Unremarkable CSF parameters included negative PCR’s for herpes simplex I and II virus, Epstein Barr virus, cytomegalovirus, varicella zoster and toxoplasma as well as negative bacterial cultures, cryptococcus antigen, and cytology examination for malignant cells. Transcranial magnetic stimulation showed an increased latency between the primary motor cortex and the left arm, whereas the right arm and both legs showed normal conduction. Serologic investigation of onconeural antibodies showed a highly positive titer for anti-Ri (1:2560, normal <80). The positive indirect immunofluorescence technique (IIFT) was validated with immunoblotting, which substantiated the isolated presence of the Ri-antibodies (Figure 2). As a result of this the diagnosis paraneoplastic brainstem encephalitis was established. An intravenous methylprednisolone therapy with 500 mg for 5 days led to rapid improvement of the patients neurological symptoms with concomitant decline of the anti-Ri titer from 1:2560 to 1:320. On follow-up 3 months after steroid therapy, the patient is currently completely asymptomatic. A regular methylprednisolone therapy for 5 days intravenously every 8 weeks is planned over a treatment period of at least 2 years. Since brainsteam encephalitis as paraneoplastic neurological syndrom have not been described in patients with metastatic rectum NEN before, other tumors were ruled out including a normal gynecological examination and a FDG-PET-CT without evidence for a lung tumor.

## Conclusion

### Rectum NET

Approximately 5-20% of all gastrointestinal neuroendocrine neoplasms (NEN) are localized in the rectum [3, 7]. In 50% the diagnosis of a rectal NEN is made upon routine endoscopy for colorectal cancer screening [8]. More frequent usage of colonoscopic screening programs might explain the increasing incidence in recent years. Most commonly, in 90% of the cases, rectal neuroendocrine neoplasms are diagnosed in a localized stage [9]. Metastatic spread is rare at the time of diagnosis, adding up to 2-10% [2, 10, 11]. Localized rectal NEN have a 5-year survival rate of 90%. In contrast, distant metastatic spread is associated with a 5-year overall survival rate of 30% [9] and a median survival of 22 months [11]. Negative prognostic factors for malignant behavior are tumor size, depth of invasion, lymph node involvement and higher histopathological grading (proliferation rate >2% and mitotic index ≥ 2/10 HPF) (Table 1) [2, 12–14]. Tumor size > 20 mm is associated with elevated risk of distant metastases and poorer prognosis [3, 15]. Lymphovascular invasion likely represents another risk factor [10, 12]. Our patient had a well differentiated tumor with a Ki-67 index of less than 2% and a tumor diameter of less than 1 cm. None of the above mentioned criteria predicting malignant behavior were present (Table 1). Nevertheless the patient was amongst the 5% of patients with NEN of the rectum which are diagnosed with distant hepatic metastasis. The median survival rate for this patient population averages 22 months. Our patient remarkably exceeds this median survival more than 5-fold (Table 2). She clearly benefited from TACE treatment resulting in disease stabilization for many years with excellent quality of life until the paraneoplastic symptoms occurred.

**Table 1:** **Factors predicting malignant behavior (8, 11)**

Predictive factor	
Tumor size	> than 20 mm
Mitotic index	>2/10 high power fields
Muscularis layer invasion	--
Lymphovascular invasion	--

**Table 2:** **Epidemiologic data (6)**

	**Incidence**
Neuroendocrine tumor	5,25/100.000
Neuroendocrine tumor of the rectum	0,86/100.000
**Rectal NET**	**General data (our patient)**
Age diagnosis years	56(40)
Disease stage at diagnosis (%)	
Localized	92
Regional	4
Distant	5
Survival rate (months)	
Localized	290
Regional	90
Distant	22(132)

### Paraneoplastic neurological syndromes

Paraneoplastic syndromes form a heterogeneous group of complications associated with malignancy that are caused neither by local effects of the tumor mass or its metastases, nor by vascular, infectious, or nutritional impairments. In several literature reports, paraneoplastic syndromes are estimated to occur in approximately 0.01 to 8% of cancer patients [16, 17]. The most frequent cancer entity associated with paraneoplastic syndromes is small cell lung cancer [5] followed by breast cancer, gynecologic tumors and hematologic malignancies such as lymphoma [4]. A paraneoplastic syndrome can affect various organs and has been proposed to be caused by two main pathophysiological mechanisms. First, tumors are capable of producing a variety of functionally active peptides that imitate hormone function and lead to a metabolic disturbance (as in endocrine paraneoplastic syndromes) [18]. In neurologic paraneoplastic syndromes it has been described that tumors ectopically express antigens that are normally expressed in the nervous system which leads to an immune-mediated cross-reactivity [19, 20]. Neurologic PNS may involve any part of the nervous system (central, peripheral or the neuromuscular junction) [21]. The production of antibodies against tumorous antigens is an autoimmune process [22]. The similarity of tumor antigens to elements of the nervous system leads to an attack of tumor-directed antibodies against nerval epitopes. These antibodies are known as onconeural antibodies and are commonly used in the diagnostic work-up to diagnose paraneoplastic neurological syndromes [4]. The diagnostic criteria for a PNS include the presence of cancer within the next 5 years, the definition of a classical or non-classical syndrome and the presence of well-defined onconeural antibodies [23]. Based on the classification in classic and non-classic PNS, brainsteam encephalitis is affiliated to the non-classic forms.

There are various cases in the literature describing patients with neuroendocrine carcinoid tumors and neurological symptomes with the tumors being localized in the stomach [24] or the bronchial system [25, 26]. In the majority of these cases the neurological affections were due to the hormones produced by the carcinoid or by the metastases [27]. Only a minority of single cases accomplish the diagnostic criteria of a PNS (e.g. positive antibodies). One case of PNS in neuroendocrine tumor of the rectum has been reported in association with anti-Hu antibodies [28]. To our knowledge, our case represents the first describing a *non-*functional neuroendocrine tumor leading to a paraneoplastic neurological syndrome due to anti-Ri antibodies. The Anti-Ri antibody which was found in our case belongs to the group of antibodies whose strong association to cancer has been proven. Most commonly this antibody is associated with breast cancer and small cell lung cancer [6, 29]. Typically, jaw dystonia and laryngospasm which were predominant symptoms of our patient are strongly associated with brainstem encephalitis due to Ri antibodies [30]. There are reports about Anti-Ri associated PNS in neuroendocrine tumors [25, 31] but to the best of our knowledge none was associated with nonfunctional rectal neuroendocrine tumors. Most effective treatment of the PNS is tumor specific treatment that is in accordance to the existing treatment guidelines of the tumor entity [32]. As described in other publications [33], immunomodulatory or –suppressive treatment leads to improvements of the functional ability of the patient but does not represent causal therapy. Our patient received high-dose immunmodulatory therapy and fortunately showed positive response. After being free from neurological symptomes the therapy was gradually reduced. Until now, there was no relapse of symptomes. Since the patient is still stable on follow-up according to imaging and biochemical means 6 months after the occurrence of the PNS, so far we have no indication that the PNS represents an early sign of disease recurrence.

In summary, this report represents the first case of an anti-Ri positive PNS occurring in a non-functional rectal NEN. Neurological symptoms in a patient with NEN should always trigger further work-up to rule out a PNS also in patients who are long-term clinically stable.

## Materials and methods

We retrospectively analyzed a patient with metastasized neuroendocrine rectum neoplasm treated in our institution since 2003. This case presentation was conducted in accordance with the Declaration of Helsinki and with the approval of the local ethics committee at the University of Marburg. The evaluation of the patient-related information was done with patient informed consent. Tumor tissue was explored immunohistochemically concerning expression of Chromogranin, Synaptophysin, Ki-67. Analyses were performed according to a standardized protocol using Leica-Bond-Max-Autostainer and the antibodies in the following dilutions: Chromogranin: Dako 1:2000; Synaptophysin: Dako 1:50; Ki-67: Dako 1:1000. For indirect immunofluorescence technique (IIFT) patient serum in various dilutions (1:10-1:5120) was incubated with tissue sections of primate cerebellum, nerve and gut (Neurology Mosaik1, Fa. Euroimmun, Lübeck). Fluorescein-tagged goat-anti-human-IgAGM detected bound anti-Ri antibodies. The IIFT was corroborated via immunoblotting. 1.5 ml of serum (dilution 1:101) was incubated with the following antigen fragments: Amphiphysin, CV2, PNMA2 (Ma2/Ta), Ri, Yo and Hu (Euroline Profil 2, Fa. Euroimmun Lübeck). Alkaline phosphatase-labelled goat-anti-human-IgG served as the enzyme conjugate.

## Consent

Written informed consent was obtained from the patient for publication of this Case report and any accompanying images. A copy of the written consent is available for review by the Editor of this journal.

## Authors’ information

Patrick Michl and Sebastian Krug shared last authorship.
